# BIOLOGICAL AGING AS A MEDIATOR OF DEPRESSION AND MORTALITY

**DOI:** 10.1093/geroni/igae098.2666

**Published:** 2024-12-31

**Authors:** Kai Wei, Xiaotong Chen

**Affiliations:** Guizhou Provincial People’s Hospital, Guiyang, Guizhou, China (People’s Republic); Jing’an District Centre Hospital of Shanghai, Shanghai, Shanghai, China (People’s Republic)

## Abstract

**Background:**

Depression has been linked to death from all-cause, cardiovascular disease and cancer; however, the underlying mechanisms remain unclear. We aimed to examine the mediating role of biological aging in the association of depression with mortality in a representative sample of the US population.

**Methods:**

Longitudinal data were obtained from 33,434 participants aged ≥20 years in the 2005-2018 cycle of the National Health and Nutrition Examination Survey and their mortality data through 2019. The Patient Health Questionnaire (PHQ-9) was used to measure depressive symptoms, and major depression was defined as a PHQ-9 score ≥10. Three measures of aging, including phenotypic aging acceleration (PA-Accel), anthropometric aging acceleration (AA-Accel), and Klemera and Doubal method-based biological aging acceleration (KDBA-Accel), were calculated. Weighted multiple linear regression model, Cox proportional hazards model, and mediation analysis were performed.

**Results:**

The major depression were positively associated with biological aging acceleration (PA-Accel β=1.34, KDBA-Accel β=0.88, AA-Accel β=0.36, all p < 0.0001). The mediation proportion of biological aging acceleration in associations of major depression with all-cause mortality were 21.53% (PA-Accel, p=0.0005), 14.63% (KDBA-Accel, p=0.0006), and 11.36% (AA-Accel, p=0.0004), respectively. Similar results were found in the Health and Retirement Study. Conclusions: Accelerated aging partially mediated the associations of major depression all-cause mortality in US populations.

